# Burden of Outdoor Air Pollution in Kerala, India—A First Health Risk Assessment at State Level

**DOI:** 10.3390/ijerph120910602

**Published:** 2015-08-28

**Authors:** Myriam Tobollik, Oliver Razum, Dirk Wintermeyer, Dietrich Plass

**Affiliations:** 1 Department of Environment and Health, School of Public Health, Bielefeld University, Bielefeld, Universitätsstraße 25, Bielefeld 33615, Germany; 2 Federal Environment Agency, Section Exposure Assessment and Environmental Health Indicators, Corrensplatz 1, Berlin 14195, Germany; E-Mails: dirk.wintermeyer@uba.de (D.W.); dietrich.plass@uba.de (D.P.); 3 Department of Epidemiology and International Public Health, School of Public Health, Bielefeld University, Bielefeld, Universitätsstraße 25, Bielefeld 33615, Germany; E-Mail: oliver.razum@uni-bielefeld.de

**Keywords:** Air pollution, particulate matter, environmental burden of disease, Years of Life Lost (YLL), Kerala, India

## Abstract

Ambient air pollution causes a considerable disease burden, particularly in South Asia. The objective of the study is to test the feasibility of applying the environmental burden of disease method at state level in India and to quantify a first set of disease burden estimates due to ambient air pollution in Kerala. Particulate Matter (PM) was used as an indicator for ambient air pollution. The disease burden was quantified in Years of Life Lost (YLL) for the population (30 + years) living in urban areas of Kerala. Scenario analyses were performed to account for uncertainties in the input parameters. 6108 (confidence interval (95% *CI*): 4150–7791) of 81,636 total natural deaths can be attributed to PM, resulting in 96,359 (95% *CI*: 65,479–122,917) YLLs due to premature mortality (base case scenario, average for 2008–2011). Depending on the underlying assumptions the results vary between 69,582 and 377,195 YLLs. Around half of the total burden is related to cardiovascular deaths. Scenario analyses show that a decrease of 10% in PM concentrations would save 15,904 (95% *CI*: 11,090–19,806) life years. The results can be used to raise awareness about air quality standards at a local level and to support decision-making processes aiming at cleaner and healthier environments.

## 1. Introduction

Air pollution is a well-known risk factor causing human ill-health. It is responsible for thousands of premature deaths, particularly in South Asia [1]. Considering global levels of ambient particulate matter (PM), India ranks tenth and thus is among the most polluted countries with an annual average PM_10_ level of 134 μg/m^3^. 42 Indian cities are listed among the 100 most polluted cities worldwide [2]. According to the latest update of the Global Burden of Disease (GBD) 2010 study 627,426 (95% *CI*: 528,681–726,434) deaths were caused by ambient PM pollution in India. Combining mortality and morbidity effects of air pollution, and using the Disability-Adjusted Life Year (DALY) as a measure for population health, 17,760,000 healthy life years (95% *CI*: 15,201,700–20,705,000) were lost in India in 2010. Most of the DALYs were lost due to mortality effects (95.6%) with only 4.4% attributable to outcomes of morbidity [1].

To estimate the health risk of air pollution an indicator needs to be defined that approximates the level of air pollution. One of the best studied indicators are PM_10_ (coarse particles smaller than 10 µm in aerodynamic diameter) and PM_2.5_ (fine particles smaller than 2.5 µm in aerodynamic diameter) [3,4]. PM is a mixture of small components and thus not a homogeneous stressor and its composition varies by location and sources [5,6,7]. The respirable fraction of PM consists mainly of organic and elemental carbonaceous materials; inorganic components such as sulfate, nitrate, and ammonium; and metal components such as iron, aluminum, nickel, copper, zinc, and lead. Coarse particles comprise primarily organic and elemental carbon and metals such as silicon, magnesium, iron, ions like sulphates, nitrates, and ammonium [8]. In India the main anthropogenic sources of PM are road traffic emissions, industrial combustion plants, commercial, and residential combustion such as cooking with solid fuels, and agricultural activities [8]. Additional regional sources are road dust, waste burning, and sea salt [9]. 

Exposure to PM can cause several adverse health effects, including mortality and morbidity outcomes [5]. The exposure to PM is associated with an increased health risk when inhaling fine particles. Once inhaled, these particles can harm the cardiovascular system by inflammation or coagulation. Additionally, the respiratory system can be harmed, because PM can trigger pulmonary oxidative stress [6].

Based on scientific evidence regarding adverse health consequences of air pollution the World Health Organization (WHO) recommends an annual mean of not more than 20 µg/m^3^ and 10 µg/m^3^ for PM_10_ and PM_2.5_, respectively [3]. To prevent and control air pollution, India issued the Air Prevention and Control of Pollution Act in 1981 and developed National Ambient Air Quality Standards (NAAQS) to regulate pollutant emissions. In 2009, the standards were updated and 12 air pollutants are currently regulated. The annual mean standards for PM_10_ and PM_2.5_ are 60 and 40 µg/m^3^ [10].

To tackle the air pollution problem, regional assessments are necessary especially in countries such as India where large health and environmental disparities exist. The differences between states in terms of population, climate, and air pollution are large and need to be considered in state-specific assessments. Such adapted risk assessments can help to raise awareness for ambient air pollution and the resulting health risks. Furthermore, they can support policy and programs and help to introduce measures to reduce ambient air pollution [11,12,13].

For our purpose, we focus Kerala, a state on the southern tip of the Indian subcontinent with a coastline of about 580 km. In 2011, Kerala had 33.4 million inhabitants (16.0 million males and 17.4 million females). Nearly half of these people reported living in urban areas (47.7%). With a sex ratio of 1084 women per 1,000 men, Kerala has the highest share of females in the population among all Indian states [14].

In this study we aim to test the feasibility of the environmental burden of disease at state level in India. In addition, we quantify a first set of disease burden estimates due to ambient air pollution in urban areas of Kerala.

## 2. Data and Methods

### 2.1. Quantification Method

In our study, the disease burden of ambient air pollution was quantified by the mortality component (Years of life Lost) of the DALY. The DALY measure generally combines morbidity (Years Lived with Disability-YLDs) and mortality (Years of Life Lost-YLLs) in one measure and thus allows comparisons of different diseases, interventions, populations, and periods [15,16].

The YLLs were calculated using the Environmental Burden of Disease (EBD) approach, an extension of the burden of disease approach, which was developed by WHO, the World Bank and the Harvard School of Public Health [17,18]. The number of deaths in a certain age-group attributable to ambient PM exposure was multiplied with the remaining life expectancy at the age of death. The deaths attributable to PM exposure were calculated as a population attributable fraction (PAF) and suitable concentration-response functions [17]. The concentration-response functions are available with a confidence interval (CI) and the upper and lower bounds were used to calculate the CI of the YLLs. The PAF was calculated with this formula [19,20]:

(1) $$PAF=\frac{\sum_{i=1}^{n}P_{i}\left(RR_{i}-1\right)}{\sum_{i}^{n}P_{i}\left(RR_{i}-1\right)+1}$$

The calculations were performed in an Excel environment using predefined and adapted spreadsheets as published by the WHO [21]. Uniform age-weights and no time-discount were applied for the estimates. To account for the state-specific setting, the life expectancies from the urban population of Kerala was used [22].

### 2.2. Data Input

Several datasets are needed to perform the calculation of EBD [17]. Table 1 summarizes the input data used in this study.

**Table 1:** Input data used for the calculation of EBD due to ambient air pollution in urban Kerala.

Data	Reference area	Source	Reference Year	Stratified by		
				Age	Sex	Rural/ Urban
PM data	Measured data for six cities in Kerala	CPCB [23]	2008–2011	–	–	Urban only
Concentration-response function for PM and all-cause mortality/cardiovascular mortality	Meta-analyses based on studies from the U.S.A., Germany, the Netherlands, Switzerland, Canada, China and New Zealand	Hoek *et al.* [24]	1976–2008 (range of the follow-up period in the meta-analyses)	Applicable only for people aged 30 years and older	–	–
	Four cities in northern China	Zhang *et al.* [25]	1998–2009	Applicable only for people aged 30 years and older	Yes	–
Population data	Kerala	Government of India [14]	2011	Yes (1 year age groups)	Yes	Yes
Life table	Kerala	Registrar General India [22]	2006–2010	Yes (1 year age groups)	Yes	Yes
Cause specific mortality data	Kerala (coverage only 12.2% of total deaths)	Office of the registrar general, India [26]	2010	Yes (10 years age groups)	Yes	–
Mortality data	Kerala (no ICD for cause of death)	Office of the registrar India [27]	2011	Yes (10 years age groups)	Yes	Yes

ICD: International Classification of Diseases, a standard diagnostic tool to classify diseases.

#### 2.2.1. Particulate Matter Data

PM_10_ is measured all over India. It is monitored in an eight-hour sampling twice a week, which results in 104 observations per year [8]. For Kerala, these data were available on the internet (open access) in form of regularly published reports by the Central Pollution Control Board (CPCB) [23]. Table 2 shows the data available for Kerala, which were based on 17 measurement sites in six cities. Therefore only the burden of diesease of the urban population can be assessed.

**Table 2:** Annual mean PM_10_ concentration (in μg/m^3^) measured by CPCB at six locations in Kerala from 2008 to 2011, Source [23].

City	Number of Stations	2008	2009	2010	2011
Kochi	7	43	42	36	38
Kozhikode	2	34	32	42	46
Thrissur	1	–	–	31	33
Mallapuram	1	–	–	39	30
Trivandrum	4	67	61	56	58
Kollam	2	–	–	47	53

For the quantification of the disease burden attributable to air pollution an annual mean value of 44.9 µg/m^3^ PM_10_ was calculated by taking into account all measured values from 2008 to 2011 to smooth out annual outliers. The most current evidence on concentration-response functions relate to PM_2.5_ instead of PM_10_ but for Kerala comprehensive PM_2.5_ data were not available, therefore, PM_10_ measurements were converted into PM_2.5_ by using recommendations of the WHO [3] and two recent studies from India [23,28] suggesting PM_2.5_ to PM_10_ ratios of 0.4, 0.5, and 0.7.

Furthermore, a counterfactual value was needed to assess the negative health effects above the comparative exposure concentration. For PM_2.5_ so far no threshold was identified below which no negative health effects of PM_2.5_ occur [3,29]. Nevertheless zero pollution is not a realistic assumption due to natural sources of PM_2.5_. Therefore we used two different counterfactual levels: (a) a theoretical minimum exposure of 7.3 µg/m^3^ derived from the largest cohort study on air pollution in the United States of America [29,30,31]; and (b) the guideline value of 10 µg/m^3^ as recommended by the WHO air quality guidelines [3].

#### 2.2.2. Concentration-Response Functions

Two concentration-response functions were used in this assessment. One is from an international meta-analysis because it was assumed that pooled results of different studies increases the consistency and validity of the concentration-response function. The other is from a Chinese cohort study because the exposure situation in China is rather comparable with the one in India (Table 3). For clear assignment of concentration-response functions and health data, International Classification of Disease 10 (ICD-10) codes were used.

**Table 3:** Selected concentration-response functions for all-cause mortality and cardiovascular mortality and PM_2.5_/PM_10_ exposure.

Source	All-Cause Mortality (ICD-10: A00-R99)	Cardiovascular mortality (ICD-10: I00-I99)	Unit
Hoek *et al.* [24]	1.062 (95% *CI*: 1.04–1.083)	1.11 (95% *CI*: 1.05–1.16)	per 10 μg/m^3^ change in PM_2.5_
Zhang *et al.* [25]	1.24 (95% *CI*: 1.22–1.27)	1.23 (95% *CI*: 1.19–1.26)	per 10 μg/m^3^ change in PM_10_

#### 2.2.3. Population Data

Population data were obtained from the Indian census 2011 [32]. The data are stratified by states, sex, five-year age groups, and the rural-urban status. Life tables for Kerala with reference years from 2006 to 2011 are also available from the census [22].

#### 2.2.4. Mortality Data

For mortality data, two data sources were used. For the EBD calculation only natural deaths (ICD 10: A00-R99) and for the scenario analysis only cardiovascular deaths (ICD 10: I00-I99) are needed. Therefore data from the Report on Medical Certification of Cause of Death 2010 [26] and the Vital statistics of India based on the Civil Registration System 2011 [27] were combined by applying the cause of death rates to the total number of deaths. These data are stratified by states, sex, five-year age groups) and urban-rural status.

Table 4 shows the demographic data for the urban population of Kerala in the year 2010. From the about 33.4 million inhabitants 7,610,740 men and 8,307,037 women lived in urban areas. The largest numbers of individuals are in the age group 0 to 14 years (676,030 boys and 647,412 girls). A sex difference is visible with 696,297 more women in the total population. The opposite distribution can be found in the mortality data. More men than women died in 2010 (48,292 men and 33,346 women). Most of the deaths occurred in older age groups (age group 70+:5727 per 100,000 men and 3,872 per 100,000 women, respectively). 

**Table 4:** Demographic data of the population living in urban areas of Kerala in 2010, stratified by age groups and sex, Sources: [14,22,26,27].

**Age**	**Population**		**Natural Deaths**		**Natural Deaths per 100,000 People**		**Cardiovascular Deaths**		**Cardiovascular Deaths per 100,000 People**	
	M	W	M	W	M	W	M	W	M	W
<1	116,460	113,490	1,679	1,286	1442	1133	41	36	35	32
1–4	477,265	459,537	227	173	48	38	22	10	5	2
5–9	616,113	590,795	133	105	22	18	14	13	2	2
10–14	676,020	647,412	146	115	22	18	15	15	2	2
15–19	632,095	612,644	395	232	62	38	75	29	12	5
20–24	618,543	661,205	386	250	62	38	73	32	12	05
25–29	564,939	670,117	680	355	120	53	128	88	23	13
30–34	535,243	641,998	644	340	120	53	121	84	23	13
35–39	553,478	681,566	1,296	658	234	97	304	145	55	21
40–44	539,207	627,153	1,262	606	234	97	296	134	55	21
45–49	527,161	593,487	3,291	1,338	624	225	853	300	162	51
50–54	446,274	480,709	2,786	1,084	624	225	722	243	162	51
55–59	414,667	424,358	5,633	2,423	1,359	571	1,666	712	402	168
60–64	333,759	355,965	4,534	2,032	1,359	571	1,341	597	402	168
65–69	218,695	258,174	5,679	3,438	2,597	1,332	1,871	1,346	856	521
70+	340,821	488,427	19,519	18,911	5,727	3,872	7,185	7,784	2,108	1,594
Total	7,610,740	8,307,037	48,290	33,346	635	401	14,727	11,569	194	139

#### 2.2.5. Scenario Analyses

To reflect existing uncertainties in the input data, several input parameters were altered: two concentration-response functions, three PM_2.5_ to PM_10_ ratios, and two counterfactual values were used to estimate the impact of parameter changes on the EBD estimates. Combining the different options resulted in ten scenarios (Table 5). The baseline scenario (*Natural Deaths (ND)_Baseline (1))* summarizes the assumption of a conservative concentration-response function, the mid value of the PM_2.5_ to PM_10_ ratio, and the theoretical minimum risk exposure as counterfactual value.

**Table 5:** Parameter scenario descriptions by considered concentration-response functions, PM_2.5_ to PM_10_ ratios, and counterfactual values. ND: Natural Deaths, CD: Cardiovascular Deaths.

Scenario	Concentration-Response Function (per 10 μg/m^3^)	PM_2.5_ to PM_10_ Ratio	Counterfactual Value in μg/m^3^
Natural deaths excluding accidents (ICD 10: A00–R99)			
ND_Baseline (1)	1.062 (95% *CI*: 1.040–1.083) ^a^	0.5 ^c^	7.3 ^e^
ND_Low PM_2.5_ to PM_10_ ratio (2)	1.062 (95% *CI*: 1.040–1.083) ^a^	0.4 ^d^	7.3 ^e^
ND_High PM_2.5_ to PM_10_ ratio (3)	1.062 (95% *CI*: 1.040–1.083) ^a^	0.7 ^d^	7.3 ^e^
ND_Alternative counterfactual value (4)	1.062 (95% *CI*: 1.040–1.083) ^a^	0.5 ^c^	10 ^c^
ND_Alternative CRF (5)	1.24 (95% *CI*: 1.22–1.27) ^b^	-	20 ^c^
Deaths caused by diseases of the circulatory system (ICD 10: I00–I99)			
CD_Baseline (6)	1.11 (95% *CI*: 1.050–1.16) ^a^	0.5 ^c^	7.3 ^e^
CD_Low PM_2.5_ to PM_10_ ratio (7)	1.11 (95% *CI*: 1.050–1.16) ^a^	0.4 ^d^	7.3 ^e^
CD_High PM_2.5_ to PM_10_ ratio (8)	1.11 (95% *CI*: 1.050–1.16) ^a^	0.7 ^d^	7.3 ^e^
CD_Alternative counterfactual value (9)	1.11 (95% *CI*: 1.050–1.16) ^a^	0.5 ^c^	10 ^c^
CD_Alternative CRF (10)	1.23 (95% *CI*: 1.19–1.26) ^b^	–	20 ^c^

^a^ Hoek *et al.* [24], ^b^ Zhang *et al.* [25], ^c^ WHO [3], ^d^ Satsangi *et al.* [28], ^e^ Lim *et al.* [31].

Air pollution is not a constant environmental factor and the counterfactual values used for the quantification are currently not achievable in India, therefore two additional and more realistic assumptions were considered: a possible decrease and increase in PM_2.5_ by 10% each (Table 6). 

**Table 6:** Air pollution scenario descriptions by considered concentration-response functions, PM_2.5_ to PM_10_ ratios, counterfactual values, and assumptions on the development of PM.

Scenario	Concentration-Response Function (per 10 μg/m^3^)	PM_2.5_ to PM_10_ Ratio	Counterfactual Value in μg/m^3^	Assumption (PM_2.5_ Development)
Natural deaths ICD 10: A00-R99				
ND_10% increase in PM_2.5_ (11)	1.062 (95% *CI*: 1.040–1.083) ^a^	0.5^c^	7.3 ^e^	10% less PM_2.5_
ND_10% decrease in PM_2.5_ (12)	1.062 (95% *CI*: 1.040–1.083) ^a^	0.5^c^	7.3 ^e^	10% more PM_2.5_
Deaths caused by diseases of the circulatory system ICD 10 I00-I99				
CD_10% increase in PM_2.5_ (13)	1.11 (95% *CI*: 1.050–1.16) ^a^	0.5 ^c^	7.3^e^	10% less PM_2.5_
CD_10% decrease in PM_2.5_ (14)	1.11 (95% *CI*: 1.050–1.16) ^a^	0.5 ^c^	7.3^e^	10% more PM_2.5_

^a^ Hoek *et al.* [24], ^c^ WHO [3], ^e^ Lim *et al.* [31].

## 3. Results and Discussion

### 3.1. Results

In the recent years (2008–2011), the annual mean PM_10_ concentrations in ambient air in urban areas of Kerala did not exceed the national guideline value of 60 μg/m^3^—except for two values in Trivandrum in 2008 and 2009 which were slightly above the guideline value (Table 2). However, the measured values were considerably higher than the guidelines recommended by WHO (20 µg/m^3^ PM_10_).

**Figure 1:**
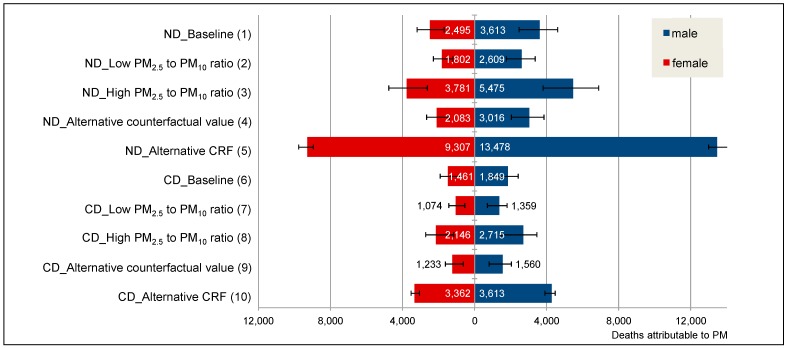
Deaths attributable to air pollution (PM) by different scenarios for the male and female urban population of Kerala. ND: Natural Deaths, CD: Cardiovascular Deaths.

In the baseline scenario (*ND_Baseline (1)*), 6,108 (*CI*: 4150–7791) of the 81,636 total natural deaths in the urban population of Kerala can be attributed to ambient air pollution by PM_2.5_ (Figure 1). Hence 7.5% of deaths can be attributed to PM_2.5_. Stratified by sex and in absolute numbers, more attributable deaths were modeled for men with 3,613 (*CI*: 2455–4609) deaths, as compared to 2495 (*CI*: 1695–3183) deaths for women. According to the assumptions in the different scenarios the results vary markedly. The lowest burden in terms of premature deaths was estimated for scenario *ND_High PM_2.5_ to PM_10_ ratio (3)*, using the more conservative value for the conversion factor from PM_2.5_ to PM_10_ (0.4). The highest burden can be found in scenario *ND_Alternative CRF (5)*, with 22,785 (*CI*: 21,912–23,909) attributable deaths.

The scenario analysis shows that many deaths which can be attributed to ambient air pollution by PM_2.5_ are due to cardiovascular causes. In the baseline scenario (*Cardiovascular Deaths (CD)_Baseline (6)*) 51% of the male and 49% of the female cardiovascular deaths can be attributed to air pollution. The sex difference was smaller than for the total natural deaths. The lowest number of premature death cases occur in scenario *CD_Low PM_2.5_ to PM_10_* *ratio (7)* and the highest in scenario *CD_Alternative CRF (10).*

Comparable patterns of disease burden were estimated for YLLs attributable to ambient PM_2.5_ (Table 7). In the baseline scenario (*ND_Baseline (1)*) for total natural deaths, 96,359 (*CI*: 65,479–122,917) life years were lost due to PM_2.5_. The absolute burden was higher in the male population with 58,868 (*CI*: 40,003–75,094) YLLs compared to 37,490 (*CI*: 25,476–47,823) YLLs in the female population. Per 100,000 people 605 (*CI*: 411–772) years of life are lost. Scenario *ND_Low PM_2.5_ to* *PM_10_* *ratio* *(2)* and *ND_High PM_2.5_ to PM_10_ ratio* *(3)* show the impact of the change in the conversion factor from PM_2.5_ to PM_10_. If a conversion factor of 0.4 was applied, the burden was calculated to 69,582 (*CI*: 46,873–89,463) YLLs. If the ratio was 0.7, the burden more than doubles to 146,020 (*CI*: 100,860–183,589) YLLs. Scenario *ND_Alternative counterfactual value* *(4)* represents the results of using a counterfactual value of 10 µg/m^3^ PM_2.5_, with 80,434 (*CI*: 54,375–103,085) YLLs. These results are lower compared to the baseline scenario because adverse health effects below 10 µg/m^3^ PM_2.5_ were not included. The highest burden was estimated for scenario *ND_Alternative CRF* *(5)* with 359,465 (*CI*: 345,695–377,195) YLLs, which is more than 3.5 times that of the baseline scenario. 

**Table 7:** YLLs and YLLs per 100,000 inhabitants due to PM_2.5_ in urban areas of Kerala, stratified by sex, *CI* in parentheses.

Scenario	YLLs			YLLs per 100,000		
	Men	Women	Total	Men	Women	Total
ND_Baseline (1)	58,868	37,490	96,358	773	451	605
	(40,003–75,094)	(25,476–47,823)	(65,479–122,917)	(526-987)	(307–576)	(411–772)
ND_Low PM_2.5_ to PM_10_ ratio (2)	42,510	27,072	69,582	559	326	437
	(28,636–54,656)	(18,237–34,807)	(46,873–89,463)	(376–718)	(220–419)	(294–562)
ND_High PM_2.5_ to PM_10_ ratio (3)	89,208	56,812	146,020	1172	684	917
	(61,619–112,160)	(39,242–71,429)	(100,861–183,589)	(810–1,474)	(472–860)	(634–1,153)
ND_Alternative counterfactual value (4)	49,139	31,294	80,433	646	377	505
	(33,219–62,977)	(21,156–40,107)	(54,375-103,084)	(436–827)	(25–483)	(342–648)
ND_Alternative CRF (5)	219,608	139,857	359,465	2,885	1684	2258
	(211,195–230,440)	(134,500–146,755)	(345,695–377,195)	(2775–3028)	(1619–1767)	(2172–2370)
CD_Baseline (6)	28,086	19,880	47,966	369	239	301
	(14,637–36,706)	(10,361–25982)	(24,998–62,688)	(192–482)	(125–313)	(157–394)
CD_Low PM_2.5_ to PM_10_ ratio (7)	20,639	14,609	35,248	271	176	221
	(10,520–27,4717)	(7,447–19,407)	(17,367–46,824)	(138–360)	(90–234)	(113–294)
CD_High PM_2.5_ to PM_10_ ratio (8)	41,235	29,188	70,423	542	351	442
	(22,376–52,394)	(15,839–37,087)	(38,215–89,481)	(294–688)	(191–446)	(240–562)
CD_Alternative counterfactual value (9)	23,688	16,768	40,456	311	202	254
	(12,184–31,257)	(8,624–22,125)	(20,808–53,382)	(160–411)	(104–266)	(131–335)
CD_ Alternative CRF (10)	64,608	45,732	110,340	849	551	693
	(58,899–68,061)	(41,691–48,176)	(100,590–116,237)	(774–849)	(502–580)	(632–730)

In scenario analyses, which specifically estimate the disease burden for cardiovascular diseases in the baseline scenario (*CD_Baseline (6)*) 47,966 (*CI*: 24,998–62,688) years of life are lost due to ambient PM_2.5_ pollution. As in the natural deaths calculation, a sex difference is visible with more male YLLs.

**Figure 2:**
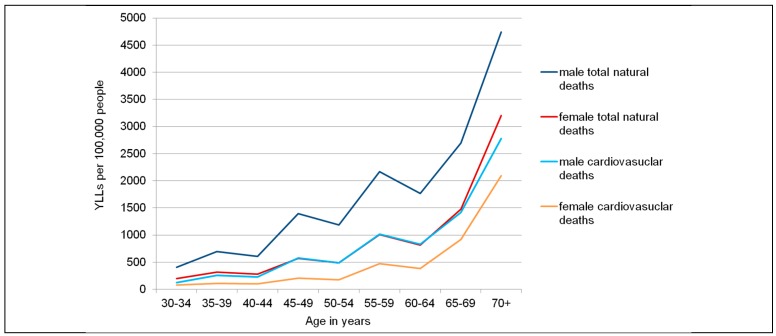
Age patterns of YLLs per 100,000 people due to PM in the baseline scenarios (*ND_Baseline (1) and CD_Baseline (6)*), in urban Kerala.

The age pattern of the disease burden in the baseline scenario (*ND_Baseline (1)*) is shown in Figure 2. For both sexes the disease burden is increasing throughout the age-groups, with some minor decreases. The highest burden in natural deaths is in the oldest age group, with over 27% of YLLs in men and even 42% of YLLs in women. The cardiovascular death burden is also highest in the oldest age group. 

**Figure 3:**
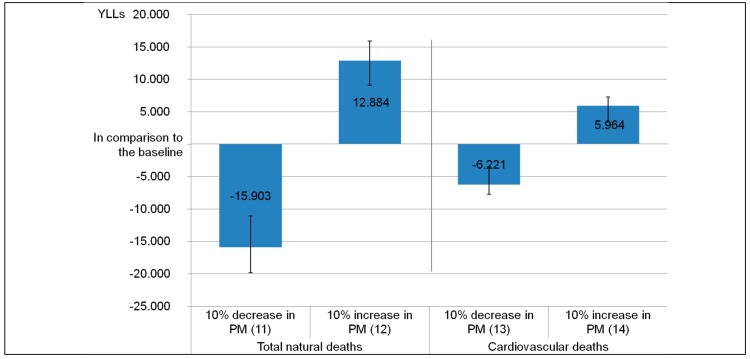
Impact on the burden of disease in urban Kerala of 10% less and 10% more PM_2.5_ compared to the baseline scenario, scenarios 11 to 14.

Assuming that air pollution and thus the PM_2.5_ concentration levels will change in the future, Figure 3 shows the impact of a 10% decrease of PM_2.5_ (*ND_10% decrease in PM_2.5_* *(12)* and *CD_10% decrease in PM_2.5_ (14)*) and a 10% increase *ND_10% increase in PM_2.5_ (11)* and *CD_10% increase in PM_2.5_ (13)*). Improved air quality regarding PM_2.5_ would reduce the burden by 15,904 (*CI*: 11,090–19,806) to 80,455 (*CI*: 54,389–103,111) YLLs as compared to the baseline scenario (*ND_Baseline (1)*). In scenario *CD_10% increase in PM_2.5_ (13)*, 41,745 (*CI*: 21,519–54,992) YLLs still can be attributed to air pollution by PM_2.5_, which would be 5954 (*CI*: 3479–7696) YLLs less compared to the cardiovascular baseline scenario (*CD_Baseline (6)*). 

A worsening of air quality by 10% more PM_2.5_ would increase the burden to 109,242 (*CI*: 74,547–13,826) YLLs. In total 12,884 (*CI*: 9068–15,909) YLLs more would occur due to higher PM_2.5_ concentrations. The cardiovascular burden would increase to 53,930 (*CI*: 28,405–69,951) YLLs. 

### 3.2. Discussion

The aim of the study was to test the feasibility of the environmental burden of disease approach at state level in Kerala, India, and to quantify a first set of disease burden estimates due to ambient air pollution by PM_2.5_. In general, despite some limitations in data availability, the method was applicable at state level. The disease burden due to ambient air pollution for the urban population was estimated to the best of our knowledge for the first time, using state specific data such as PM_10_ concentrations, population and mortality data. Data on air pollution were freely available, but the locations of the single measurement sites were missing as well as exposure data for the rural population. Therefore, population-weighted exposure modeling was not possible. Nevertheless, the results show the importance of air pollution as a threat to population health in Kerala.

The ambient PM_10_ values in Kerala did not exceed the Indian national guidelines. However, these standards are lagging far behind other national and international standards [9]. Our results support this criticism by showing the burden of PM_2.5_. Further, realistic future scenarios of PM_2.5_ were assessed, demonstrating that a worsening of air pollution (a 10% increase in PM_2.5_) would increase the mortality-associated disease burden by around 13%. By improving air quality (a 10% decrease in PM_2.5_), around 17% of the disease burden attributed to PM_2.5_ could be prevented. 

The scenario analysis shows that around half of the natural deaths which can be attributed to PM_2.5_ are due to cardiovascular causes (*ND_Baseline (1)* and *CD_Baseline (6)*): natural deaths 6108 (*CI*: 4150–7791) and cardiovascular deaths 3,311 (*CI*: 1725–4327) attributable to air pollution). The other 50% of the natural deaths may have other causes like lung cancer, chronic obstructive pulmonary disease, or other respiratory diseases.

In our assessment, we used air pollution data published by CPCB of India. Comparing these data to other sources provides some differences in the EBD results. The national annual average of PM_10_ concentration in ambient air in India from 2009 to 2012 was 132 µg/m^3^ [33]. This value is much higher than the value we used for our calculations (44 µg/m^3^ PM_10_). In the last GBD study a weighted annual mean PM_2.5_ of 27.2 µg/m3 was used to quantify the burden of air pollution in India [34,35], which is in the range of PM_2.5_ we applied (18.0 to 31.5 µg/m^3^ PM_2.5_). Data from the same source extracted for Kerala provide an even lower value of 14.5 µg/m^3^ PM_2.5_. This population weighted mean refers to the state of Kerala in total and thus includes rural areas [35]. 

In the scenario analyses different PM_2.5_ to PM_10_ ratios were assessed because so far PM_2.5_ is not comprehensively measured in India and no agreed and exact ratio is available. The ratios applied in our study vary from 0.4 to 0.7 [23,28]. The results differ accordingly: when applying a ratio of 0.4, the burden is 69,582 (*CI*: 46,873–89,463) YLLs for natural causes, which is around half of the burden when applying a ratio of 0.7 (146,020 (*CI*: 100,860–183,589). 

The disease burden estimates are a first set of results and should be interpreted with caution. No estimates for Kerala were available so far, therefore estimates from India and South Asia were used to compare the results. In the recent burden of disease estimates published by WHO, the premature deaths attributable to ambient air pollution in South East Asia were 52 per 100,000 persons for 2012 [36]. In our study, 38 natural deaths per 100,000 people (*CI*: 26*–*49) can be attributed to ambient air pollution in Kerala. However, considering the much broader reference area in the WHO estimates, the differences are reasonable—particularly so as the WHO applied much higher PM level values than we did in our assessment.

In the GBD 2010 study the mortality part of disease burden of ambient PM in India was estimated to be 1,358 (95% *CI*: 1192*–*1617) YLLs per 100,000 people, which is much higher than our estimate (605 (*CI*: 411–772) YLLs per 100,000 people). Reasons for the large differences could be the different input data. In the GBD 2010 study, many more deaths were considered in the calculation (896 male and 771 female deaths per 100,000 for India and our numbers are 635 males and 401 females deaths per 100,000 for Kerala) and different concentration-response functions and life expectancies were applied because in the GBD study an international comparison was targeted [31]. Another reason for the comparably low disease burden calculated in the present assessment could be that nearly half of the population in Kerala was living in urban areas, but only around 34.4% of deaths were reported there.

Because of the complex and data-demanding calculations, our study also faces some limitations, mostly related to data quantity and quality. The best available data were used, but still limitations and uncertainties occur which are discussed in the following.

No comprehensive data on the cause of death were available. Therefore, data from the Report on Medical Certification of Cause of Death 2010 and the Vital statistics of India based on the Civil Registration System 2011 were combined, while keeping in mind that the combination of two data sources can lead to several uncertainties. Data on causes of death were classified in 10-year age groups. In order to enable reliable quantifications it was necessary to distribute these data to five-year age groups using population data. This does not give an accurate distribution of the death causes. Nevertheless, for the assignment of the remaining life expectancy to the age groups and the quantification of the YLLs it is sufficiently detailed. 

The highest available age group in the mortality data is 70 years and older. In this age group the disease burden caused by PM is the highest for natural deaths as well as for cardiovascular deaths with respect to absolute numbers of premature deaths. More detailed data for the age groups older than 70 years would allow more accurate results because there is evidence that elderly people are more susceptible to the effects of air pollution [37]. Additionally, young children, undernourished people, and those with pre-existing health problems should be considered in more detail because they would benefit most from better air quality [38,39]. 

Beside the uncertainties related to the PM exposure, the conversion factor from PM_10_ to PM_2.5_ and mortality data, the applicability of concentration-response functions is questionable. Over the last decades a growing number of experimental and epidemiological studies increased the knowledge of the association between PM exposure (especially PM_2.5_) and adverse health effects [3,4,24,40], but evidence concerning the statistical relationship, which is needed for an health risk assessment, is still limited, especially for India [13]. During the last few years, two time series studies for Delhi and Chennai were conducted to assess the link between PM exposure in ambient air and the number of natural deaths. However, these results cannot be directly used in this assessment, because the study focused on short-term exposure solely [41]. So far no cohort studies on long term exposure to air pollution and mortality have been reported for India [42]. Therefore, and because of broad consistency of Asian time-series studies with European and North American studies, the Health Effects Institute (HEI) supports the use of results from Western cohort studies, if data for estimating the burden of disease attributable to air pollution in Asia is missing [42]. 

Nevertheless, this approach has limitations because the concentration-response functions were derived at lower levels of air pollution than observed in Asia and thus the results must be interpreted with caution. Therefore, two concentration-response functions were used in this assessment to show the impact of this input variable. Comparing scenario *ND_Alternative counterfactual value* with an excess risk of 6% (95% *CI*: 4%–8%) per 10 µg/m^3^ increase in PM_2.5_ exposure with scenario *ND_Alternative CRF* and a 24% (95% *CI*: 22%–27%) excess risk per 10 µg/m^3^ PM_10_ increase, shows a difference of 279,031 (*CI*: 291,321–274,110) YLLs. Thus, applying the concentration-response function estimated for China resulted in a burden four times as high as when using the pooled concentration-response function. These large differences prove the strong influence of the concentration-response function on the EBD calculation and the need to research on concentration-response functions for India and other South Asian countries. Compared to the other input variables the choice of the concentration-response function has the largest effect on the results.

Several other adverse health effects of exposure to PM are discussed, but convincing evidence is still lacking [4,40]. As soon as sufficient evidence is available, further disease endpoints need to be included in the estimation processes to better illustrate and underline the importance of air pollution as a major health threat. Additionally the related health data (mortality and morbidity) is needed to assess the effects of PM comprehensively. If, for example, prevalence data on cardiovascular and respiratory health outcomes would be available, the morbidity part (years lived with disability) could be quantified as well. 

The approach presented here can be adopted by other Indian states by applying respective population and ambient air pollution data. However, the availability of concentration-response functions should be examined because, compared to other Indian states, the air pollution levels in Kerala are relatively low. Thus if necessary, an adapted concentration-response function for higher air pollution levels and another slope (e.g. supralinear) should be applied to avoid an overestimation [43]. Finally, the assessment needs to be further developed in the direction of an integrated approach by including rural settings as well as indoor exposure as suggested by Balakrishnan, Dhaliwal and Shah [12].

#### 3.2.1. Implication for Further Research

- Conduct a cohort study to assess the effects of long-term air pollution exposure on health outcomes (mortality and morbidity) and to derive representative concentration-response functions for Indian settings.
- Expand the number of measurement parameters of air pollution, like PM_2.5_, to provide more specific and reliable data for health risk assessments. Likewise, the number of measurement sites should be increased to also cover rural areas. This would allow a much more comprehensive risk assessment.
- Assess indoor air pollution as well and include measurements in the YLL estimations at state level.

#### 3.2.2. Practical Implications

The identification of the sources of air pollution is another step to develop effective mitigation policies. India’s development over the last decades is characterized by a social and economic progress which is directly linked to industrialization, urbanization, and motorization, all leading to an increase in ambient air pollution. This development will most probably continue in the future [44]. In particular, the demand for personal transport and the amount of goods transportation are increasing steadily [45]. This in turn leads to an increase of pollutant emissions from vehicle exhausts. Therefore, actions to reduce hazardous emissions are needed, such as fuel emission standards or a shift to a safer and cleaner public transport alternative [9]. Because public transport is not an option for everybody, actions addressing the road conditions and the traffic itself are needed. Poor road conditions, the high number of vehicles, waterlogging during monsoons, and people on the street interrupt the traffic and lead to traffic congestions, which in turn can increase the fuel consumption [44]. Better road maintenance, paving of unpaved roads, and silt removal would be possible actions. The low quality of fuel and lubricating oil currently used also contributes to poor air quality [44]. CPCB is already discussing a road map for fuel quality improvement in India. 

Further pollution sources are solid fuels used for cooking and industrial emissions. The latter need to be regulated by appropriate policies, for example by a shift from coal based industry to the use of cleaner and renewable fuels such as wind or solar energy. A shift to cleaner fuel is also needed for cooking, because indoor air pollution causes a considered health burden [12].

## 4. Conclusions

Our findings show that the EBD method is applicable at state level and can be applied to other Indian states as well. The results indicate, that even if local air quality standards are met, a considerable health burden for the population living in urban Kerala can be assumed, which can be partly prevented by taking actions to reduce air pollution. Compared to other Indian states Kerala shows relative low annual PM levels, thus the burden of disease due to PM in other Indian states is expected to be even higher. Further estimates for other Indian states can help to complete the overall picture and allow for state-wise comparisons.

## References

[1] Global Burden of Disease Study 2010 (GBD 2010) Results by Risk Factor 1990–2010—Country Level. http://ghdx.healthdata.org/record/global-burden-disease-study-2010-gbd-2010-results-risk-factor-1990-2010-country-level.

[2] Ambient Air Pollution Database. http://www.who.int/phe/health_topics/outdoorair/databases/cities-2011/en/.

[3] WHO, (World Health Organisation) (2006). Air Quality Guidelines Global Update 2005.

[4] Brook R.D., Rajagopalan S., Pope C.A., Brook J.R., Bhatnagar A., Diez-Roux A.V., Holguin F., Hong Y., Luepker R.V., Mittleman M.A. (2010). Particulate matter air pollution and cardiovascular disease: An update to the scientific statement from the american heart association. Circulation.

[5] Samet J.M., Brauer M., Schlesinger R., World Health Organization (2006). Particulate matter. Air Quality Guidelines. Global Update 2005: Particulate Matter, Ozone, Nitrogen Dioxide and Sulfur Dioxide.

[6] Anderson J.O., Thundiyil J.G., Stolbach A. (2012). Clearing the air: A review of the effects of particulate matter air pollution on human health. J. Med. Toxicol..

[7] WHO (2013). Health Effects of Particulate Matter. Policy implications for countries in Eastern Europe, Caucasus and Central Asia.

[8] National Ambient Air Quality Status & Trends in India—2010. http://www.cpcb.nic.in/upload/NewItems/NewItem_192_NAAQSTI.pdf.

[9] Guttikunda S.K., Goel R., Pant P. (2014). Nature of air pollution, emission sources, and management in the Indian cities. Atmos. Environ..

[10] The Gazette of India Revised National Ambient Air Quality Standards (NAAQS) 2009, Part III—Section 4. http://www.cpcb.nic.in/upload/Latest/Latest_48_FINAL_AIR_STANDARD.pdf.

[11] Frequently Asked Questions: Ambient and Household Air Pollution and Health Update 2014. http://www.who.int/phe/health_topics/outdoorair/databases/faqs_air_pollution.pdf.

[12] Balakrishnan K., Dhaliwal R.S., Shah B. (2011). Integrated urban-rural frameworks for air pollution and health-related research in India: The way forward. Environ. Health Perspect..

[13] Yamamoto S.S., Phalkey R., Malik A.A. (2014). A systematic review of air pollution as a risk factor for cardiovascular disease in South Asia: Limited evidence from India and Pakistan. Int. J. Hyg. Environ. Health.

[14] Census of India 2011, Primary Census Abstract, Data highlights Kerala, Series 33. http://www.censusindia.gov.in/2011census/PCA/PCA_Highlights/pca_highlights_file/kerala/Data_highlights.pdf.

[15] Murray C.J.L., Salomon J.A., Mathers C.D., Murray C.J.L., Salomon J.A., Mathers C.D., Lopez A.D. (2002). A critical examination of summary measures of population health. Summary Measures of Population Health; Concepts, Ethics, measurements and Applications.

[16] Knol A.B., Petersen A.C., van der Sluijs J.P., Lebret E. (2009). Dealing with uncertainties in environmental burden of disease assessment. Environ. Health.

[17] Prüss-Üstün A., Mathers C., Corvalán C., Woodward A. Introduction and Methods: Assessing the Environmental Burden of Disease at National and Local Levels. http://www.who.int/quantifying_ehimpacts/publications/en/leadebd2.pdf.

[18] Summary Measures of Population Health: Concepts, Ethics, Measurement and Applications. http://www.jstor.org/stable/41110882?seq=1#page_scan_tab_contents.

[19] Martuzzi M., Mitis F., Iavarone I., Serinelli M. (2006). Health Impact of PM_10_ and Ozone in 13 Italien Cities.

[20] Ezzati M., Lopez Alan D., Rodgers A., Murray C.J.L. Comparative Quantification of Health Risks: Global and Regional Burden of Disease Attributable to Selected Major Risk Factors. http://www.who.int/publications/cra/en/.

[21] Health Statistics and Information Systems: National Tools. http://www.who.int/healthinfo/global_burden_disease/tools_national/en/.

[22] SRS Based Abridged Life Tables 2003–07 to 2006–10. http://www.censusindia.gov.in/vital_statistics/SRS_Based/Cover_Page.pdf.

[23] Annual Report 2011–2012. http://cpcb.nic.in/upload/AnnualReports/AnnualReport_43_AR_2011-12_English.pdf.

[24] Hoek G., Krishnan R.M., Beelen R., Peters A., Ostro B., Brunekreef B., Kaufman J.D. (2013). Long-term air pollution exposure and cardio-respiratory mortality: A review. Environ. Health.

[25] Zhang L.W., Chen X., Xue X.D., Sun M., Han B., Li C.P., Ma J., Yu H., Sun Z.R., Zhao L.J. (2014). Long-term exposure to high particulate matter pollution and cardiovascular mortality: A 12-year cohort study in four cities in northern China. Environ. Int..

[26] Report on Medical Certification of Cause of Death 2010. http://www.censusindia.gov.in/2011-Documents/mccd_Report1/MCCD-Report-2010.pdf.

[27] Vital Statistics of India Based On the Civil Registration System 2011. http://www.censusindia.gov.in/2011-Documents/CRS_Report/CRS%20Report_2011.pdf.

[28] Satsangi P.G., Kulshrestha A., Taneja A., Rao P.S.P. (2011). Measurements of PM_10_ and PM_2.5_ in aerosols in Agra, a semi-arid region of India. IJ RSP.

[29] Burnett R.T., Pope C.A., Ezzati M., Olives C., Lim S.S., Mehta S., Shin H.H., Singh G., Hubbell B., Brauer M. (2014). An integrated risk function for estimating the global burden of disease attributable to ambient fine particulate matter exposure. Environ. Health Perspect..

[30] Krewski D., Jerrett M., Burnett R.T., Ma R., Hughes E., Shi Y., Turner M.C., Pope C.A., Thurston G., Calle E.E. Extended Follow-Up and Spatial Analysis of the American Cancer Society Study Linking Particulate Air Pollution and Mortality. http://scientificintegrityinstitute.net/Krewski052108.pdf.

[31] Lim S.S., Vos T., Flaxman A.D., Danaei G., Shibuya K., Adair-Rohani H., Amann M., Anderson H.R., Andrews K.G., Aryee M. (2012). A comparative risk assessment of burden of disease and injury attributable to 67 risk factors and risk factor clusters in 21 regions, 1990–2010: A systematic analysis for the global burden of disease study 2010. Lancet.

[32] The Registrar General & Census Commissioner Population enumeration data (final population). Single year age data - c13 table (india/states/uts) Ministry of Home Affairs, Government of India.: New Delhi, 2010–11. http://www.censusindia.gov.in/2011census/population_enumeration.html.

[33] WHO, World Health Organisation Public health and environment (PHE): Ambient air pollution. Exposure to Particulate Matter Less Than 10 µm in Diamter in Urban Areas, 2008–2013. http://gamapserver.who.int/gho/interactive_charts/phe/oap_exposure/atlas.html.

[34] Balakrishnan K., Cohen A., Smith K.R. (2014). Addressing the burden of disease attributable to air pollution in india: The need to integrate across household and ambient air pollution exposures. Environ. Health Perspect..

[35] Brauer M., Amann M., Burnett R.T., Cohen A., Dentener F., Ezzati M., Henderson S.B., Krzyzanowski M., Martin R.V., Van Dingenen R. (2012). Exposure assessment for estimation of the global burden of disease attributable to outdoor air pollution. Environ. Sci. Technol..

[36] WHO, (World Health Organisation) Public Health and Environment: Ambient Air Pollution. Burden of Disease, Deaths. http://apps.who.int/gho/data/node.main.156?lang=en.

[37] WHO, (World Health Organisation) (2006). Health Effects and Risks of Transport Systems: The Hearts Project.

[38] Environmental health inequalities in Europe. Assessment Report. http://www.euro.who.int/en/publications/abstracts/environmental-health-inequalities-in-europe.-assessment-report.

[39] Nema P., Goyal S.K., Gurjar B.R., Molina L.T., Ojha C.S.P. (2010). Estimation of health impacts due to PM_10_ in major Indian cities. Air Pollution: Health and Environmental Impacts.

[40] WHO European Centre for Environment and Health (2013). Review of Evidence on Health Aspects of Air Pollution—Revihaap Project Technical Report.

[41] Public Health and Air Pollution in Asia (PAPA). Coordinated Studies of Short-Term Exposure to Air Pollution and Daily Mortality in Two Indian Cities. http://pubs.healtheffects.org/view.php?id=357.

[42] Outdoor Air Pollution and Health in the Developing Countries of Asia: A comprehensive review. http://pubs.healtheffects.org/getfile.php?u=602.

[43] Pope C.A., Cropper M., Coggins J., Cohen A. (2015). Health benefits of air pollution abatement policy: Role of the shape of the concentration-response function. J. Air Waste Manag. Assoc..

[44] Ray M.R., Lahiri T., Gurjar B.R., Molina L.T., Ojha C.S.P. (2010). Health effects of urban air pollution in india. Air Pollution: Health and Environmental Impacts.

[45] Guttikunda S.K., Jawahar P. Road Transport in India 2010–30: Emissions, Pollution, and Health Impacts. http://urbanemissions.info/india-road-transport.

